# Sex-Related Differences of Weight Bearing and Non-Weight Bearing Muscle Properties

**DOI:** 10.3390/muscles2040031

**Published:** 2023-12-15

**Authors:** Omid Nabavizadeh, Ashley A. Herda

**Affiliations:** 1 Department of Bioengineering, University of Colorado Anschutz Medical Campus, Aurora, CO 80045, USA; omid.nabavizadeh@cuanschutz.edu; 2 Department of Health, Sport, and Exercise Sciences, University of Kansas, Lawrence, KS 66046, USA

**Keywords:** muscle composition, muscle quality, ultrasound

## Abstract

This study evaluated muscle composition, quality, and strength of non-weight bearing and weight bearing muscles between males and females. Twenty-eight, healthy males (*n* = 14; mean ± SD; age = 25.1 ± 4.2 years; height = 181.9 ± 10.6 cm; weight = 91.6 ± 17.2 kg) and females (*n* = 14; age = 25.0 ± 3.4 years; height = 165.9 ± 6.9 cm; weight = 66.0 ± 10.2 kg) underwent body composition assessment to estimate body fat (%BF) and total-body, arm, and leg fat-free mass (TFFM, ArmFFM, and LegFFM, respectively) and muscle composition via B-mode ultrasound to measure muscle cross-sectional area (mCSA), echo intensity (EI), and thickness (mT) of four muscles [rectus femoris (RF), vastus lateralis (VL), flexor digitorum superficialis (FDS), and flexor carpi radialis (FCR)]. Additionally, upper- [handgrip strength (HG)] and lower-body [leg extension (LE)] maximal strength were measured, recorded, and expressed relative to FFM to determine muscle quality (MQ) for the dominant arm and leg, respectively. Males had greater TFFM, ArmFFM, and LegFFM (*p* < 0.001), mCSA for RF, VL, FCR, and FDS (*p* < 0.001), and mT for RF, VL (*p* < 0.001–0.006). Females had greater EI for RF, VL, and FDS (*p* = 0.003–0.01). Negative correlations were identified between EI and MQ for all muscles in males and females, however, no significance was determined. Despite the sex differences in absolute strength and size, muscle quality (relative strength) was not different for the upper nor lower body.

## 1. Introduction

Maximal strength of males and females typically occurs between the ages of 22 and 40 years, and subsequently tends to decrease over the next several decades, dependent on activity level through adulthood [1]. Studies have reported significant differences in skeletal muscle between males and females, which includes fiber type composition, energy metabolism, and muscular strength [2,3,4,5]. According to Glenmark et al., males have a greater anaerobic metabolism and generate higher power outputs than females [2]. Simoneau and Bouchard examined the fiber types of the vastus lateralis (VL) and noticed the fiber areas of types I, IIA, and IIB were 14, 38, and 56% larger than females [6]. Several studies examine the interaction between strength and sex in weight bearing and non-weight bearing muscle, separately [7,8,9,10] but do not compare weight bearing and non-weight bearing directly. Bouillon et al. reported maximal voluntary isometric contraction percentages were similar in weight bearing muscles when compared between sexes [7]. Another study conducted by de Boer et al. examined the muscle adaptations of both weight bearing and non-weight bearing muscles while on bed rest. It was reported that weight bearing muscles decreased after 5-weeks and no significant differences were seen in non-weight bearing muscles [8]. However, most of these studies focus on cross-sectional area (mCSA) through the use of magnetic resonance imaging (MRI), and dual-energy X-ray absorptiometry (DXA) rather than mCSA or echo intensity obtained with an ultrasound (US) scan. US can be used to non-invasively quantify muscle composition, such as mCSA and muscle quality, via echo intensity, which assesses the proportion of lean mass vs intramuscular fat infiltration as an arbitrary grey-scale value [11].

Echo intensity is a gray-scale computerized analysis, calculating the mean pixel intensity of a specific region without including subcutaneous fat and bone [12]. By projecting lighter or darker images, echo intensity has the ability to extract an arbitrary value between 0 and 256 [12]. According to Wilhelm et al. [13], echo intensity of the quadriceps was significantly correlated to that of 1-repetition maximum strength test in older men (age: 66.1 ± 4.5 years). Additionally, the amount of muscle mass can also easily and non-invasively be assessed using bioelectrical impedance analysis (BIA), to estimate fat mass, fat-free mass, and total body water in various populations [14]. Using multiple methods to estimate muscle mass (quantity and quality) will provide useful and meaningful information as to estimate of the contractile and non-contractile tissue used during strength tests. This approach has yet to be utilized in directly comparing weight bearing muscle characteristics of the lower limbs (quadriceps) to those of non-weight bearing muscles in the upper limbs (forearm).

Therefore, the purpose of this study was to evaluate muscle quality and quantity between sex in non-weight bearing and weight bearing muscle properties of younger adults. It is hypothesized that males will have greater amounts of muscle cross-sectional area and muscle thickness with greater amounts of absolute and relative strength in both non-weight bearing and weight bearing muscles when compared to females. It is also hypothesized that echo intensity will be higher in females in comparison to males due to higher amounts of intramuscular fat. Additionally, this study evaluates the differences in body composition between males and females, where it is hypothesized that males will have greater amounts of total and segmental lean mass and females will have greater amounts of percent body fat.

## 2. Results

Independent *t*-tests for body composition indicated several differences between males and females (Table 1). Specifically, males had a greater quantity of TFFM than females (mean difference: 23.87 kg, *p* < 0.001). Males also had greater quantity of LegFFM (mean difference: 2.66 kg, *p* < 0.001) and ArmFFM (mean difference: 1.97 kg, *p* < 0.001). However, there was no significant difference in %BF between males and females (mean difference: 5.99%, *p* = 0.077).

**Figure 1:**
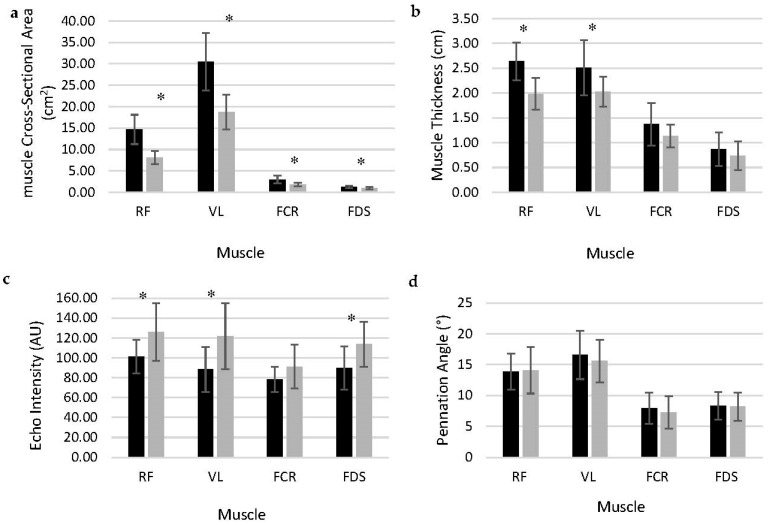
Muscle characteristics of males (black) and females (grey) for (**a**) mCSA, (**b**) mT, (**c**) EI, and (**d**) PA. mCSA: muscle cross-sectional area; mT: muscle thickness; EI: echo intensity; PA: pennation angle; RF: rectus femoris; VL: vastus lateralis; FCR: flexor carpi radialis; FDS: flexor digitorum superficialis. * Indicates significant difference between sex.

With ultrasound image analysis (Figure 1), males had larger mCSA than females for the RF and VL (Figure 1a; mean differences: 6.56, *p* < 0.001 and 11.77, *p* < 0.001, respectively) and greater mT of the RF and VL (Figure 1b; mean difference: 0.66, *p* < 0.001 and 0.50, *p* = 0.006, respectively). Alternatively, females had higher EI than males for the RF and VL (Figure 1c; mean difference: 24.70, *p* = 0.011 and 33.78, *p* = 0.003, respectively). Similar patterns were also observed for the forearm: mCSA was larger for males than females for the FCR and FDS (Figure 1a; mean difference: 1.21, *p* < 0.001 and 0.29, *p* < 0.001, respectively) and females had higher FDS EI than males (Figure 1c; mean difference: 24.21, *p* = 0.008). The FCR EI indicated no differences between sex (Figure 1c; mean difference: 10.80, *p* = 0.114), nor were there differences in mT of the FCR or FDS between males and females (Figure 1b; mean difference: 0.14, *p* = 0.326, and 0.09, *p* = 0.429, respectively). There were no differences between the two sexes for RF and VL PA (Figure 1d; mean difference: 0.20, *p* = 0.874 and 0.93, *p* = 0.509, respectively). Additionally, no differences were observed between males and females PA in FCR and FDS (Figure 1d; mean difference: 0.66, *p* = 0.502 and 0.14, *p* = 0.870, respectively).

The independent *t*-tests evaluating male and female absolute handgrip strength (HG) and maximum leg extension (LE) indicated males had greater strength than females for both variables (mean difference: 25.79 kg, *p* < 0.001 and 24.96 kg, *p* < 0.001, respectively). However, there were no differences indicated between males and females relative arm muscle quality (ArmMQ; mean difference: 0.13, *p* = 0.894) and leg muscle quality (LegMQ; mean difference: 1.03, *p* = 0.064).

### Relationships among Muscle Characteristics with Relative and Absolute Strength

All evaluated Pearson’s Product Moment Correlations (*r*) for males and females are reported in Table 2. All Pearson’s Product Moment Correlation graphs can be visualized in Supplementary Materials. Notable relationships that may lead to future prediction of relative or absolute strength from muscle characteristics include total armFFM for males [b-coefficient = 0.815 (SE = 1.765) *p* < 0.001] to predict HG strength and none identified for females. No lower body variables were identified to predict LE strength in males, however, one was identified for females [legFFM: b-coefficient = 0.652 (SE = 2.03) *p* = 0.012]. Due to multicollinearity, none of the muscle quality variables were identified.

## 3. Discussion

The primary purpose of this study was to identify any differences between sex for muscle characteristics, such as mCSA, EI, PA, and mT of upper and lower body muscles. Additional aims included the evaluation of sex differences in overall body composition, absolute and relative strength of the upper and lower body and any relationships among muscle characteristics and performance. Additionally, exploratory regression analysis was used to determine the best predictor, if any, of upper and lower body strength, separately, in males and females. Overall, the primary results indicated that males had 57.5% greater mCSA in the RF, 47.8% greater in the VL, 50% greater in the FCR, and 26.5% greater in the FDS when compared to females. These larger mCSAs throughout the body are reported as the primary contributor to greater overall body mass and absolute strength for males [15]. Whereas females had higher EI for RF, VL, and FDS (Table 1) when compared to males. mT was 28.6% greater in the RF and 22.1% greater in the VL for males, however, no significant differences were seen between sexes for mT in FCR and FDS. Additionally, PA of the various muscles were not notably different between males and females.

Males had significantly greater mCSA for RF, VL, FCR, and FDS. These findings are not surprising due to the copious amounts of literature stating similar findings [16,17,18]. However, many studies use MRI, CT, or muscle biopsy to identify fiber and mCSA, not musculoskeletal US. Stock et al., measured 20 male and female college students examining the differences between regional and total skeletal muscle of the VL and RF. Results were similar to the current study as significant differences in mCSA were reported between males and females for both the VL and RF (mean differences: 3 cm^2^ and 2.8 cm^2^, respectively) [18]. This may be due to greater fiber area and larger fiber size which would increase the mCSA shown in skeletal muscle for males [4,6].

Additionally, females had significantly greater EI for RF, VL, and FDS than males. This may be due to a higher concentration of intramuscular fat content and architectural features in the muscle fascicles [19,20]. Reimers et al. [21], examined 86 muscle biopsies and measured EI and intramuscular fat content and concluded that increases in EI occurred due to the amount of intramuscular fat within the muscle, changing the acoustics of the ultrasound waves. Several other studies also reported high EI for females when compared to males [20,22]. According to Egan and Zierath [23], higher intramuscular fat is associated with type-I muscle fibers. Taking this into consideration, females are reported to have a greater percentage of type-I muscle fibers compared to type-II [24], which coincides with increases of intramuscular fat content. Even though EI for FCR was not significant (*p* = 0.114), females still had higher EI compared to males (mean ± SD arbitrary units (AU); 88.9 ± 21.1 AU and 78.1 ± 12.7 AU, respectively). Witteveen et al. [25], conducted a study examining the differences between FCR EI of male patients with intensive care unit-acquired weakness (ICU-AW) to patients without ICU-AW. Results were similar to the present study and showed no significant differences in male patients with ICU-AW to those without ICU-AW in the FCR EI (mean difference arbitrary units (AU): 5.4 AU).

Surprisingly, the current study indicated no significant differences in PA for RF, VL, FCR, and FDS (*p* = 0.874, 0.509, 0.502, and 0.870, respectively). Bartolomei et al. [26] also reported no significant differences for VL between males and females. In contrast, Kubo et al. [27], examined the PA of VL, medial gastrocnemius (MG), and triceps brachii (TB) between 311 young males and females as well as aging males and females. Results showed younger males had greater PA for all muscles when compared to females (*p* < 0.001, for all). Even though PA was not significant in the current study, we believe that motor unit action potential could play a role in strength differences. However, this was not directly measured. According to Trevino et al. [28], speculations of increased muscle fiber size translates to an increase in motor unit size, in turn, causing greater mCSA as well as strength changes. Another factor may be level of training between males and females. The use of a mixed cohort of trained individuals and lack of difference in US-acquired PA in the present sample was unique. Specifically, Coratella et al. [29] states that heavy resistance training (strength and hypertrophy) is associated with increases in pennation angle, whereas power training results in no changes. The individuals included in the current study reported to have a mix between heavy resistance training with high-speed power lifting potentially resulting no drastic differences in pennation angle [26].

As anticipated, males had significantly greater TotalFFM, LegFFM, and ArmFFM, however, no significant difference was indicated in %BF between the two sexes. Similar results can be seen by Janssen et al. [17], after examining skeletal muscle mass in 468 males and females using MRI. A main factor that may play a role in the differences between lean mass and sex differences is hormonal variations [3]. Testosterone has been shown to increase lean mass by promoting hypertrophy of myofibers within the muscle, and a subsequent reduction in fat mass [30,31]. Further, females are consistently reported with greater FM (and %BF) than to males [32]. Even though the current study did not have significant differences in %BF, females continued to have higher values.

The overall strength differences between males and females were similar for HG and LE (mean difference: 25.8 kg and 25.0 kg, respectively). These results are similar to those reported by Bishop et al. [33], who monitored the differences in upper and lower body strength of male and female swimmers and non-swimmers for handgrip and leg extension assessments. Similar results for strength differences of HG and LE with a 48.6% and 51.5% percent absolute strength difference were reported between male and female swimmers and 61.8% and 64.4% percent absolute strength difference, respectively, in non-swimmers. Though Bishop et al. [33] had not indicated why these similarities were discovered, several studies state that strength per unit of mCSA or lean mass do not differ between sexes [34,35].

There are reported differences in the neural activation patterns between isometric and isotonic forces [36]. Further, Mcguigan et al. [37] examined the relationships between isometric and dynamic strength testing in trained males. A strong correlation was determined between peak force of an isometric mid-thigh pull and 1RM back squat as well as 1RM bench press [37]. In the current study, mode does not seem to differ in terms of absolute strength, however, examining two different limbs with different methods of force production (isometric vs. isotonic) cannot be directly examined. Future studies should ensure a consistent method is used (such as isometric dynamometry for quadriceps leg extension and biceps curls) to enable more direct comparisons of strength for weight bearing and non-weight bearing muscles of males and females.

Correlations of VL EI and LegMQ, in the present study, revealed that there is a strong negative correlation, where the lower EI, the greater LegMQ. This is supported by Cadore et al. [38] that reported that connective and adipose tissue in the muscle is associated with dynamic strength. Moderate negative correlations were observed, yet not significant between RF EI and LegMQ for both males (*r* = −0.414) and females (*r* = −0.522). The current study shows nearly 17.1% and 27.2% of RF EI is related to the relative strength in males and females, respectively. VL EI and LegMQ indicated weak negative correlations in males (*r* = −0.244) and a moderate negative correlation in females (*r* = −0.477). Results showed nearly 6% and 22.7% of VL EI is related to relative strength in males as well as females. In agreement with the present study, Cadore et al. [38] also reported a negative correlation between the RF and three isokinetic peak torques in aging males (*r* = −0.48 to −0.64, *p* < 0.001). Further, Fukumoto et al. [39] also reported similar results with negative correlations between quadriceps and strength (*r* = −0.40, *p* < 0.001). EI also showed a significant negative correlation with muscle strength (*p* < 0.01). These data suggest that muscle quantity and quality independently contribute to muscle strength (Fukumoto et al., 2012). Uezumi et al. [40] expressed that amount of adipose tissue within the muscle is affected by mesenchymal stem cells (MSC). Female donor MSC have shown more vascular cell adhesion than male donor MSC [41]. This could be why females have greater amounts of adipose tissue, affecting muscle quality. Even though there has been evidence of the differences of muscle characteristics in males and females, those studies were not utilizing the use of the ultrasound images to evaluate the composition of the muscle.

For the forearm, no observed differences were seen in FCR EI. This may be because the FCR increases stiffness and stability in the joint, allowing for greater handgrip strength values [42]. However, a moderate negative correlation was examined for FDS EI and ArmMQ for males (*r* = −0.433) and a weak negative correlation in females (*r* = −0.112). Results indicated roughly 18.7% of FDS EI is related to relative strength. Fat free mass has been considered an important factor in determining relative strength [26]. In contrast, Song et al. [43] examined echo intensity as physiological marker for muscle quality. Results showed EI in arms 1 (average = 63 AU) and 2 (average = 62.2 AU) as well as legs 1 (average = 37.3 AU) and 2 (average = 36.9 AU) between males and females was not influential on muscle size and strength. FDS EI does seem to be associated with greater levels of isometric strength because the FDS is most effective when the dynamometer is in contact with the middle phalanx and can produce increases in initial strength for a short duration of time [44]. Use of ultrasound as an accurate yet non-invasive measure of muscle size and PA has not been previously utilized in this context. Further, the direct comparison between weight bearing and non-weight bearing has not been made previously.

There are several limitations to this study. One consideration that may influence the study outcomes could be the heterogeneity of the measured sample. Training levels (experience, consistency, and method) among participants make these results generalizable to recreational younger adults but perhaps not those with training-specific muscular adaptations as the literature suggests (i.e., greater pennation angle) [27]. Like many studies, sample size is a limitation for the present study as well as a lack of familiarization to the strength measurements. The data collection for this study was condensed to one study visit due to COVID-19 protocol restrictions. To counter this, the participants had some practice of the strength measurements with additional warm-up sets. Lastly, the present study includes the evaluation of two separate forces (isometric and isotonic), limiting the differentiation between weight bearing and non-weight bearing muscles. There has been limited evidence of changes in muscle properties of those two groups but no cross-over evaluation of weight-bearing and non-weight bearing muscle characteristics and relationships.

## 4. Materials and Methods

### 4.1. Study Design

This study is an observational investigation using a single visit to the Exercise and Human Performance laboratory. Participants included males and females between 18 and 38 years of age. A series of tests were completed by the participants consisting of body composition using a BIA, ultrasound (US) imaging of the dominant quadriceps and forearm muscles, and maximal strength assessment using leg extension (LE) and handgrip dynamometry (HG). The independent variables included in the study are sex and weight bearing status of muscles. Dependent variables of this study include mCSA, EI, pennation angle, maximal strength (LE and HG), and total and segmental body composition.

### 4.2. Participants

Twenty-eight healthy males (*n* = 14; mean ± SD; age = 25.1 ± 4.2 years; height = 181.9 ± 10.6 cm; body mass = 91.6 ± 17.2 kg) and females (*n* = 14; age = 25.0 ± 3.4 years; height = 165.9 ± 6.9 cm; body mass = 66.0 ± 10.2 kg) volunteered for this study. This study was approved by the university’s institutional review board for human subject research (STUDY00145497) and each participant signed an approved informed consent document prior to completing any study-related activities. A pre-determined sample size to provide 80% or greater power was identified at 8 participants per group using G* Power 3.1.9.7 [45]. Participant ID numbers were allocated as the individuals arrived at the exercise and human performance laboratory with signed consent form and complete pre assessment. Upon consent, participants also self-reported their experience and consistency with various types of aerobic, recreational, and resistance exercise.

### 4.3. Procedures

#### 4.3.1. Hydration and Body Composition Assessment

**Figure 2:**
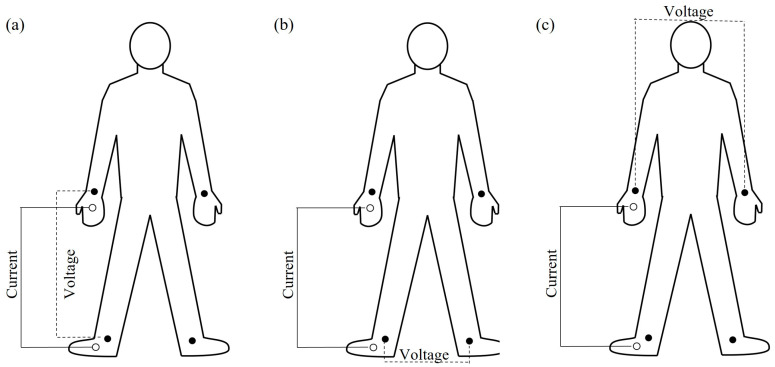
Bioelectrical impedance analysis electrode configuration for: (**a**) total body, (**b**) legs only, and (**c**) arms only.

Participants were asked to visit the laboratory in a fasted and rested state, and to refrain from caffeine and exercise 18–24 h prior to testing. Each individual was permitted to consume water ad libitum up until 1 h prior to test. Body mass (kg) and stature (cm) was assessed using a standard physician balance scale (Detecto, Cardinal, 439, Webb City, MO, USA). The BIA (ImpediMed Ltd., Pinkenba, Queensland, Australia) was used to determine segmental body composition on the dominant side of the body. Prior to electrode placement, participants were asked to provide a urine sample in a sterile urine collection cup to determine hydration status via urine specific gravity. Using a refractometer (Fisherbrand, Clinical 200 ATC, Waltham, MA, USA), the investigator used disposable pipettes to transfer three urine droplets onto the refractometer mirror. The investigator then held the refractometer up to a light source while looking through the device. A blue line indicated the participant’s urine specific gravity level through the lens and will be recorded. Individuals were subsequently asked to remove any metal jewelry, socks, and shoes before they lay supine on a padded examination table while the investigator cleans the skin from dirt, hair, or lotion with alcohol for electrode placement, reducing artifact. Electrodes were placed on each wrist at the ulnar head and between the medial and lateral malleoli of the anterior aspect on each ankle with additional electrodes placed on their right hand and foot, 5 cm distal from the first electrode’s placement to determine estimated segmental BIA. Separate scans were completed with varying electrode placement for total body arrangements (Figure 2a) and segmental (Figure 2b,c). This results in FFM (kg), percent body fat (%BF), and total arm and leg lean mass (ArmFFM; kg and LegFFM; kg, respectively). ArmFFM and LegFFM was estimated using equations from Organ et al. [46] LL^2^/R = Appendicular FFM where LL is segmental limb length and R represents resistance. Once Appendicular FFM is estimated, the number is then divided by 4 to isolate a single limb.

#### 4.3.2. Musculoskeletal Ultrasonography

Muscle composition (mCSA, EI, and muscle thickness) of the vastus lateralis (VL), rectus femoris (RF), flexor carpi radialis (FCR), and flexor digitorum superficialis (FDS) of the dominant forearm were measured using B-mode and panoramic ultrasound imaging (Logiq e, GE Healthcare, Wauwatosa, WI, USA; FDA 510[K] Number K133533). Before the measure, a generous amount of water-soluble transmission gel was applied to the skin to reduce possible near-field artifacts and enhance acoustic coupling. During the measure, the participant was asked to lay supine with the dominant-leg as well as the dominant-forearm extended and relaxed on the examination table. The investigator measured and marked 50% femur length from the lateral condyle of the knee to the greater trochanter of the femur, 10 cm superior to the superior pole of the patella, medially, and 30% from the elbow to ulnar styloid process for consistent measurements. The scan depth of 8 cm, gain was 46 dB, and transducer was 10 mHz for quadriceps and a scan depth of 4 cm, 46 dB gain, and 10 mHz transducer when looking at the forearm. An US transducer probe passed in the transverse plane to obtain panoramic scans. The US transducer probe was then held at the sagittal plane to examine pennation angle.

Each of the images were analyzed by a single investigator using imageJ (NIH, Bethesda, MD, USA) to measure mCSA, EI, sFAT, and mT. Prior to image analysis, the investigator set the scale to cm using the marks inlaid at the top of the image. To examine mT, a straight perpendicular line was drawn at the widest distance of the muscle. sFAT was measured from the bottom of the cutaneous to the muscle fascia by using the mid-point of mCSA as reference. To determine mCSA, the polygon function was used to manually trace the border of the muscle. EI was examined using the gray-scale analysis in ImageJ of the mCSA region of interest (black = 0, white = 256). sFAT measurements were used to correct EI values by using methods from Young et al. [20].

#### 4.3.3. Muscular Strength Assessment

Isometric handgrip strength of the dominant hand (kg) was then assessed using an adjustable handle hydraulic handgrip dynamometer (Jamar, Sammons Preston Roylan, Boilingbrook, IL, USA). The HG width was adjusted so the 2nd phalanx of the middle finger was perpendicular to the device and preferred hand was recorded. The participant completed the HG tests in a standing position with their arm near their body, the elbow bent at 90°, and turned similar to using a hammer. The participant was allowed several practice trials on each hand prior to a short rest and subsequent maximal measurements. The best score from three trials was recorded.

Maximal muscle strength for the upper and lower limbs was determined. Using a leg extension machine (Multi-Power Circuit, Universal Gym, Fresno, CA, USA), dominant leg strength (kg) was determined after the investigator properly demonstrates the exercise movement and required range of motion. Participants were then asked to keep their back and buttocks completely on the seat with their hands firmly gripping the handles attached to the side of the machine. After proper alignment in the device (knee in line with the axis of rotation), the participant was asked to complete full range of motion for the movement at a specific weight. Participants were given three warm-up sets to familiarize the participant with the movement on the device and sufficient rest (2–3 min) in between attempts. Each individual was then asked to complete 1-repetition at a given weight. Attempts became progressively more difficult and once the individual cannot complete 1-repetition at a given weight, the test was complete.

#### 4.3.4. Quantifying Muscle Quality

Muscle quality was determined by the ratio between strength and muscle mass. To calculate muscle quality measurements, two equations are performed. To estimate muscle quality index for the upper body (ArmMQ), HG strength (kg) was divided by total mCSA in the forearm. To determine muscle quality index of the lower body (LegMQ), 1-RM LE (kg) was divided by the total mCSA of the thigh [47]. In order to subsequently compare these values, we took the strength value of both tests and divided it by appendicular lean mass determined by the BIA.

### 4.4. Statistical Analyses

Independent *t*-tests comparing sex with Bonferroni corrections were used to analyze body composition variables from the BIA (TFFM, %BF, ArmFFM, and LegFFM), ultrasound (mCSA, EI, and mT of RF, VL, FCR, FDS). Further, muscle quality variables (ArmMQ and LegMQ) were calculated to represent relative strength of the upper and lower body. Additional Pearson correlation coefficients (*r*) were calculated to determine the relationship between pennation angles and relative strength variables for males and females. All analyses were conducted using Statistical Package for the Social Sciences (SPSS) version 27 (IBM Corp. Armonk, NY, USA) with a pre-determined alpha level of 0.05 to determine statistical significance.

## 5. Conclusions

In conclusion, males have greater muscle cross sectional area and muscle thickness. Echo intensity was greater in females than males for the VL, RF, and FDS. Additionally, no differences were observed for PA between sex. As expected, total body fat free mass, and arm and leg fat free mass were greater in males, yet there was no difference in %BF. In the present study, we observed similar strength differences in handgrip strength and leg extension strength of the dominant limbs between males and females, however, we were unable to directly compare the two. Lastly, echo intensity was negatively correlated with relative strength, while the echogenicity of the muscle image decreased, relative strength tends to increase. Despite the sex differences in absolute strength and size, muscle quality (relative strength) was not different for the upper nor lower body.

## Figures and Tables

**Table 1:** Provides the mean and standard deviation (mean ± SD), t-ratio, and effect size (Cohen’s *d*) for all the dependent variables of males and females.

	Males (*n* = 14)	Females (*n* = 14)		Effect Size
Body Composition	Mean ± SD	Mean ± SD	*t*-Ratio	Cohen’s *d*
TFFM (kg)	70.61 ± 14.24 *	46.74 ± 5.90	−5.8	2.19
% BF (%)	22.57 ± 8.95	28.56 ± 8.27	1.84	0.7
ArmFFM (kg)	4.46 ± 1.08 *	2.49 ± 0.42	−6.34	2.33
LegFFM (kg)	9.53 ± 1.90 *	6.87 ± 0.96	−4.66	1.76
**Muscle Composition Leg**				
RF mCSA (cm^2^)	14.69 ± 3.43 *	8.13 ± 1.53	−6.53	2.47
RF EI (AU)	101.53 ± 16.94	126.23 ± 29.00 *	2.75	1.04
RF MT (cm)	2.64 ± 0.38 *	1.98 ± 0.32	−4.97	1.88
VL mCSA (cm^2^)	30.52 ± 6.71 *	18.75 ± 4.07	−5.61	2.12
VL EI (AU)	88.30 ± 22.65	122.08 ± 32.14 *	3.22	1.22
VL MT (cm)	2.51 ± 0.56 *	2.01 ± 0.28	−3.02	1.14
**Muscle Composition Arm**				
FCR mCSA (cm^2^)	3.00 ± 0.87 *	1.80 ± 0.41	−4.7	1.78
FCR EI (AU)	78.12 ± 12.73	88.91 ± 21.14	1.64	0.62
FCR MT (cm)	1.37 ± 0.43	1.23 ± 0.30	−1	0.38
FDS mCSA (cm^2^)	1.24 ± 0.31 *	0.95 ± 0.26	−2.71	1.03
FDS EI (AU)	89.63 ± 21.70	113.84 ± 22.66 *	2.89	1.09
FDS MT (cm)	0.87 ± 0.34	0.78 ± 0.28	−0.8	0.3
**Relative Strength**				
ArmMQ	13.06 ± 1.90	12.94 ± 2.90	−0.14	0.79
LegMQ	7.13 ± 1.73	6.10 ± 1.00	−1.93	0.73
**Absolute Strength**				
HG (kg)	57.29 ± 11.45 *	31.50 ± 4.93	−7.73	2.93
LE (kg)	66.89 ± 18.10 *	41.94 ± 8.89	−4.63	1.75
**Pennation Angle**				
RF PA (°)	13.90 ± 2.90	14.10 ± 3.77	0.16	0.6
VL PA (°)	16.56 ± 3.90	15.62 ± 3.45	−0.67	0.25
FCR PA (°)	7.95 ± 2.51	7.29 ± 2.62	−0.68	0.28
FDS PA (°)	8.32 ± 2.25	8.18 ± 2.31	−0.17	0.06

* Indicates significant between males and females (*p* < 0.05); total fat free mass (TFFM); percent body fat (%BF); arm fat free mass (ArmFFM); leg fat free mass (LegFFM); rectus femoris (RF); vastus lateralis (VL); flexor carpi radialis (FCR); flexor digitorum superficialis (FDS); muscle cross-sectional area (mCSA); echo intensity (EI); muscle thickness (MT).

**Table 2:** Pearson correlation and level of significance for absolute and relative strength variables compared to muscle composition variables of males and females.

	Males		Females	
	Pearson’s *r*	*p*-Value	Pearson’s *r*	*p*-Value
**Leg Extension**				
RF mCSA	0.521	0.056	0.44	0.116
RF EI	−0.296	0.304	−0.373	0.188
RF mT	0.303	0.293	0.424	0.131
RF PA	−0.087	0.766	0.141	0.632
VL mCSA	0.455	0.111	0.592 *	0.026
VL EI	−0.270	0.350	−0.321	0.263
VL mT	0.402	0.154	0.202	0.488
VL PA	0.084	0.775	0.057	0.847
Total Leg mCSA	0.491	0.074	0.667 *	0.009
LegFFM	0.356	0.212	0.652 *	0.012
RF EI	−0.656 *	0.011	−0.412	0.143
VL EI	−0.407	0.148	−0.518	0.058
**Handgrip**				
FCR mCSA	0.468	0.092	0.053	0.857
FCR EI	0.340	0.235	−0.220	0.451
FCR mT	−0.071	0.810	−0.238	0.412
FCR PA	−0.293	0.310	−0.088	0.766
FDS mCSA	0.133	0.650	0.391	0.167
FDS EI	−0.427	0.128	−0.450	0.106
FDS mT	−0.115	0.697	−0.087	0.768
FDS PA	−0.133	0.651	−0.431	0.124
Total Arm mCSA	0.742 *	0.002	0.282	0.329
ArmFFM	0.815 *	<0.001	0.201	0.492
**ArmMQ**				
FCR EI	−0.050	0.865	−0.100	0.733
FDS EI	−0.413	0.142	−0.024	0.936

* Indicates a significant difference from zero correlation (*p* < 0.05); Absolute *r* values of 0.1–0.35, 0.36–0.67, 0.68–1.0 indicate weak, moderate, and strong correlations.

## Data Availability

All data pertinent to this study may be available from the corresponding author upon reasonable request.

## Supplementary Materials

The following supporting information can be downloaded at: https://www.mdpi.com/article/10.3390/muscles2040031/s1, Figure S1: Pearson Correlation Graphs for all comparisons.
