# Supplementary figures and images for: Sex-Related Differences of Weight Bearing and Non-Weight Bearing Muscle Properties
Source: Muscles. 2023 Dec 15;2(4):400–12. doi: 10.3390/muscles2040031 (PMC12225486; doi:10.3390/muscles2040031)

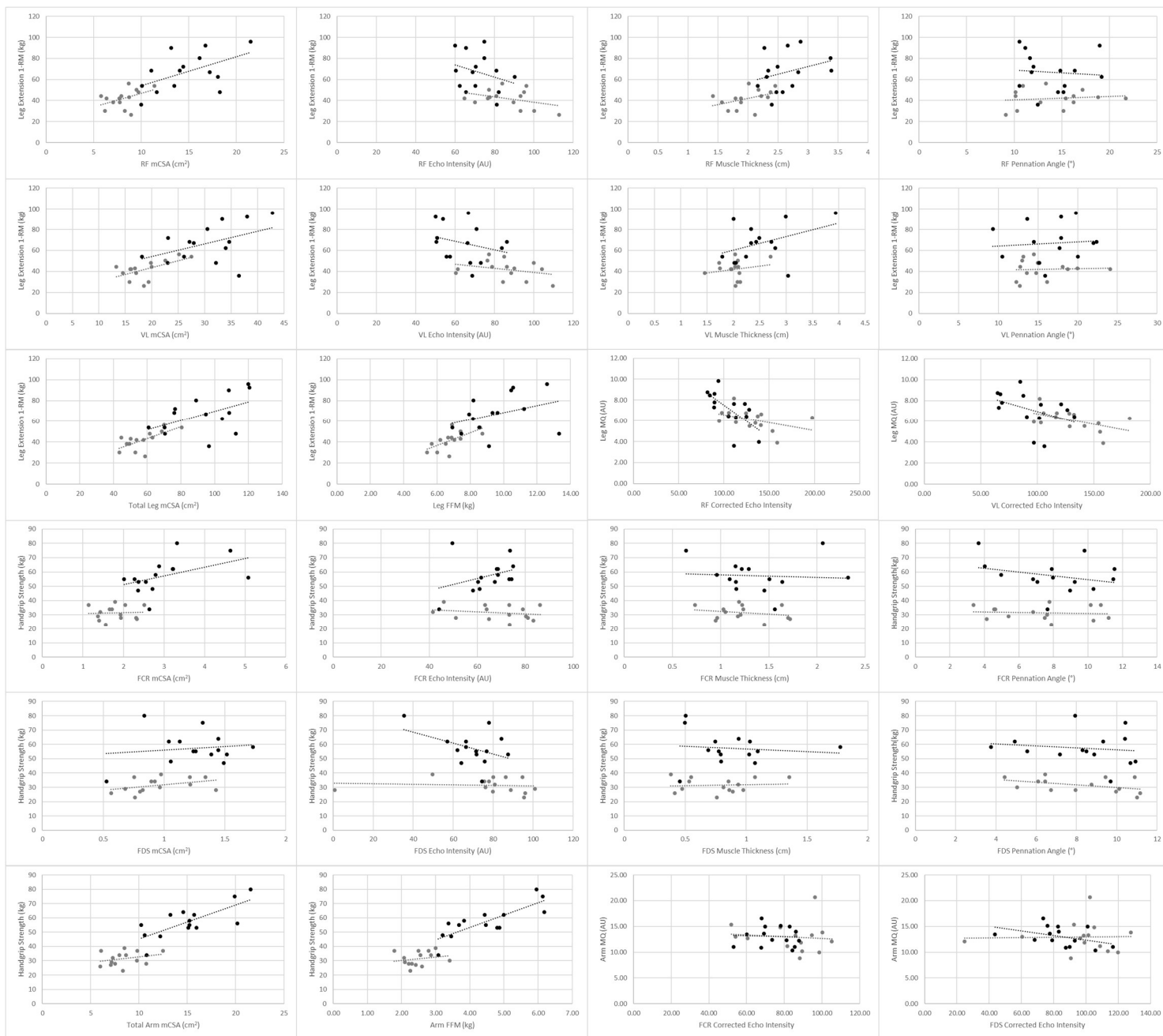

**Figure S1.** Pearson Correlation Graphs for all comparisons.

Supplement: Supplementary file 1 [file muscles-02-00031-s001.zip › muscles-2639808-supplementary.pdf]
